# Farmers’ preferences for *Peste des petits ruminants* disease vaccine delivery attributes in Uganda

**DOI:** 10.3389/fvets.2025.1715437

**Published:** 2025-12-04

**Authors:** Peter Mulindwa Lule, David Jakinda Otieno, Rose Adhiambo Nyikal, Jane Namatovu, Henry Kiara, Kristina Roesel, Emily Ouma

**Affiliations:** 1 Department of Agricultural Economics, University of Nairobi, Nairobi, Kenya; 2 International Livestock Research Institute, Kampala, Uganda; 3 International Livestock Research Institute, Nairobi, Kenya; 4 Department of Animal Breeding and Husbandry in the Tropics and Subtropics, University of Hohenheim, Stuttgart, Germany

**Keywords:** farmer preferences, *Peste des petits ruminants*, livestock production systems, vaccine attributes, discrete choice analysis

## Abstract

Small ruminants in Uganda are affected by a highly contagious disease called *Peste des petits ruminants* (PPR), commonly referred to as goat plague. It is highly contagious and has high mortality and morbidity. The disease can be controlled through vaccination, but the vaccine is not readily available and accessible in the Ugandan market, especially in the areas where the PPR problem has been identified. Furthermore, farmers tend to believe that vaccination is the mandate of the government, who should therefore bear the cost of its delivery. Therefore, the drivers of vaccination delivery become very important if the country is to eradicate PPR by 2030 as committed. This study analyzed the vaccine delivery attributes that farmers prefer in three districts of Uganda representing three production systems (pastoral, agro-pastoral and mixed crop-livestock). The study randomly sampled 444 small ruminant rearing households and used discrete choice experiments to elicit preferences. Both the random parameter logit and the latent class random parameter logit were used to analyze preferences. Farmers’ willingness to pay and the compensating surplus for different attributes and policy scenarios were estimated. The results indicated that the farmers preferred to obtain certificates after vaccination. In addition, farmers preferred closer vaccination stations to their homes and vaccination by qualified personnel. Farmers were willing to pay the highest for vaccine certification. The study recommends the provision of certificates after vaccination to allow farmers to move their animals even when quarantines are imposed as an incentive to increase vaccine coverage. Also, the participation of different stakeholders in conducting awareness campaigns for vaccination, especially with respect to the provision of quality vaccines is paramount. Lastly, the government should subsidize costs associated with administering the vaccine.

## 1 Introduction

Small ruminant production plays an important role in the livelihoods of many households around the world (1). In Uganda, livestock (including small ruminants) contribute 3.8% of the total gross domestic product (GDP) (2). As is the case within East Africa, Uganda livestock is produced under three production systems, agro-pastoral, pastoral and mixed production systems, with small ruminants mainly kept by smallholders and pastoralists (3, 4). Uganda has about 16 million goats and 4.4 million sheep with an annual growth of 2.4% (2). About 39% of the households own goats with an average herd size of five (5) animals. Small ruminants play a key role in contributing to income and food (meat and milk) for these households. They also provide manure and skin, as well as providing households with means to build on their assets. Also, small ruminants can be used to mitigate the risk of food insecurity in case of crop failure. In addition, culturally, goats play many social roles, such as exchanges during marriages, sacrifices, fines, and initiation ceremonies (4). Given the social and economic importance of small ruminants and the characteristics of the production systems, any disease entering the herd would have devastating effects on these communities (5).

One of the main diseases affecting small ruminants is a severe viral disease called *Peste des Petits Ruminants* (PPR) (6). It has been nicknamed the goat plague because the virus is related to Rinderpest virus that causes cattle plague. The PPR has high mortality rates (90–100%) and morbidity rates (50–100%) (7). Morbidity losses include weight loss in animals, an extended reproduction cycle, and a decrease in milk production in the entire herd. These epidemiological characteristics lead to households incurring increased costs when trying to control PPR (or secondary infections due to PPR) (5, 8). There have been several studies on the economic impact of PPR at the farm, village, district, and national levels. Global losses due to PPR of up to USD 2.6 billion have been reported to occur annually (8). Also, different countries have reported losses per animal. For example, Pakistan reported losses of up to USD 33 per animal, while India reported losses of USD 7.7 per animal (9, 10). In Uganda, Ayebazibwe et al. (11), estimated annual average losses due to PPR of USD 2.2 million per district in a sample of five districts. For example, in Nebbi district economic losses of USD 1.5 million (67% due to weight loss and 30% due to mortality).

In Uganda, the first PPR outbreak occurred in 2007 in the northeast of Uganda commonly called the Karamoja region (11). Currently, PPR is slowly spreading throughout the country moving from the northeast that is mainly pastoral to the central part of the country (largely mixed production system) to the southwest (mainly agro-pastoral livestock production system) (12). The pattern of spread is following a known mapped route commonly known as the cattle corridor, which stretches from North-East to South-West of Uganda (12). In this corridor, many livestock activities (production and trading) occur, precipitating the spread of PPR. Efforts have been underway to control PPR spread by government and some non-government organizations, but the task has been too complicated for several reasons.

Various measures to prevent PPR exist, including restrictions on animal movement during outbreaks, biosecurity measures (isolation of sick and new animals on the farm) and vaccination (13). Restricted movement of animals is the first control option that is applied locally, regionally and nationally in case of disease outbreak (14). Such restrictions have the advantage that they are independent of the disease, the disease-causing agent, and can therefore be enacted before the causative agent has been fully identified (14). But they are difficult to implement, especially in pastoral communities that depend on animal movement for sustainable production. However, vaccination is suggested as the main mechanism for controlling and eradicating PPR through prevention because the vaccine confers lifelong immunity. Though, vaccination is difficult to implement given mobile production systems in countries where the disease is endemic (15) Additionally, there are reports of low usage of veterinary vaccines in sub-Saharan Africa, especially among poor smallholder farmers due to financial, logistical and service delivery challenges (16, 17). Yet, the success of a good livestock disease eradication campaign requires a good vaccine, sufficient funds and appropriate policies and practices for vaccine delivery and farmer cooperation (18, 19).

In Uganda, the vaccine is not readily available and accessible in the market, especially in the rural areas where the problem has been identified. Second, the mandate for PPR vaccination is in the hands of the government, making the vaccine a public good. But the government does not have enough resources to sustainably vaccinate all small ruminants. To reduce the burden, the government has the option of allowing the vaccine to be provided to small ruminant farmers as a private good (where farmers purchase the vaccine from the private sector) coupled with an efficient vaccine delivery system as suggested by McLeod and Rushton (18). However, since the vaccine is not yet readily available in the market, as a private good farmers’ preferences and willingness to pay (WTP) for this vaccine have not yet been established. Furthermore, efficient and acceptable delivery systems have not been established, yet these act as precursors for successful vaccination campaigns as well as better establishment of the vaccine on the market. McLeod and Rushton (18), indicates cost-effective vaccination requires methods of delivery to be adapted to livestock production systems and given the diversity of these systems in Uganda, analysis is necessary. This is even further exacerbated by the belief in some production systems that vaccination is indeed the mandate of the government (20). Therefore, while broader issues like vaccine supply, distribution infrastructure and farmer perceptions of government responsibility are important, insights on farmers’ preferences of the vaccine delivery system would also improve the chances of eradicating PPR in Uganda. As such, this study set out to assess livestock farmers’ preferences for vaccine delivery inputs and their willingness to pay. The main hypothesis was that there were no differences in preference for vaccine delivery attributes within the different livestock production systems.

This study contributes to Uganda’s PPR control strategy by providing in advance of implementation, a coherent analysis of the reasoning behind the proposed control measures and the foreseeable consequences. The study provides a tool that can be used to formulate realistic strategies in implementation of PPR vaccination during the eradication campaigns.

## 2 Materials and methods

There are two methods that fit this study: contingent valuation (CV) and choice experiment (CE) approaches. Both methods have been widely used to generate potential demand for non-marketable goods with CE having higher convergent validity (21). The CV approach presents respondents with a scenario which describes a hypothetical market for a product and asks for their WTP for a good under the set of circumstances described. In contrast, the CE approach characterizes a good in terms of its main attributes and presents respondents with different sets of attribute bundles (with attributes set at varying levels) from which they choose their preferred bundle. This enables the trade-offs of the respondents between attributes to be estimated and scaled against each other. As long as price is one of the attributes, the monetary value ascribed to any individual attribute level of the good can be estimated (22). The main advantage of CE is that it can estimate these attribute values separately and not just the value of the whole good, as is the case with the CV. This study used CE as this would provide an in-depth analysis of different policy scenarios.

### 2.1 Choice experiment design

#### 2.1.1 Attribute identification

The CE design process began with the identification of relevant attributes through key informant interviews (KIIs) with relevant stakeholders. The KIIs included district officials from the veterinary department and vaccine sellers, while farmers participated in focus group discussions (FGDs). Twelve FGDs were conducted each having about 6 to 8 participants in the study sites while 12 KIIs were interviewed. Respondents in the KIIs and FGDs were asked the desirable attributes that could lead to the uptake of vaccines; how the vaccines are currently delivered to farmers and the challenges they face in the delivery of the vaccines. From these, the following attributes were generated: seasonality, thermotolerance, side effects, time of the day for vaccination, distance to vaccination centre, guaranteeing quality of the vaccine, personnel vaccinating, and ease of movement of animals after vaccination.

The attributes generated were further validated with information from other studies (19, 23–26). In these studies, some of the attributes were not suitable for a PPR vaccine study because they were more intrinsic, for example, efficacy, dosage rates and side effects. The PPR vaccine confers lifelong immunity, requires one dose, and has no side effects. Therefore, from the above studies the following attributes were considered: thermotolerance, vaccine combination and authority offering the vaccine.

#### 2.1.2 Attribute selection and level assignment

All attributes (obtained from the KIIs, FGDs and validated through literature) were then presented to two sex-segregated farmer groups (each having 10 participants), and they were ranked, using a simple pairwise ranking approach. The FGDs were sex disaggregated to capture any attributes that could be gender related. Furthermore, the intention was to enable participants to express themselves freely without intimidation from the opposite sex. Of all the attributes selected, the five with the highest rank were selected for the study and are indicated in Table 1. Appropriate levels were assigned to each, including price (Table 1). From these FGDs, farmers indicated that vaccination was always done in designated areas. The time it took farmers to take the animals to these centers was mentioned and ranked as a key factor in the vaccination campaign. The time it took to the vaccination centers was determined based on the information received during the FGDs. It was indicated that vaccination was sometimes carried out at home or some farmers had to travel distances that ranged from 30 min to an hour based on their herd sizes. The study utilized the key informants (veterinary officers and animal health service providers) to ascertain the time it took to reconstitute the vaccine when the vaccinator is carrying out a home vaccination. The key informants mentioned that it ranged between 1 and 3 min (average of 2 min). This led to the choice of the levels of 2 min indicating vaccination carried out at home, 30 min and an hour for the other two levels. Secondly, personnel vaccinating was mentioned as key due to the trust and quality issues that arise from previous vaccination experiences. This information was used to shape the attributes. Thus, an attribute based on trust (personnel vaccination) and quality (quality assurance guarantor of the vaccine) was constructed. Third, an attribute on certification of vaccinated animals was constructed to address problems of animal movement. Normally during PPR outbreaks, the only way to control the disease is by imposing quarantines (limitation of animal movement). But if the animals were vaccinated, they could hypothetically be moved during an imposed PPR quarantine. Lastly, vaccine price and administration cost attributes were added to help in determining the cost effectiveness of the vaccine (18). The administration cost included the fee for the person restraining the goat and their fuel to the farm (unitized per goat). The price per dose of vaccine was determined based on the price list from the Kenya Veterinary Vaccine Production Institute (KEVEVAPI) because it was the closest source of the vaccine to Uganda and based on information obtained from vaccine wholesalers (using their pricing strategy). Therefore, the price levels covered the lowest to the highest range that could be charged per goat/sheep. The price ranges were determined based on the study area. The highest price was determined by how much a vaccine distributor would charge if it was in a remote area, and the lowest price would be the price it would charge if it was in an urban area. This list was then presented and validated at the Uganda national PPR steering committee meeting.

**Table 1:** Attributes included in the CE design of the PPR vaccine delivery.

Attributes	Levels	Variable name
Time to vaccination center	2 min 30 min 60 min	TIME (continuous)
Quality assurance guarantor of the vaccine	Local leader Government animal health worker Neighbor that has vaccinated (reference level)	QLTL QLTD QLTN
Proof of vaccination certificate	Given certificate Not given certificate (reference level)	CERTY CERN
Personnel vaccinating	Government animal vaccinator Community animal health vaccinator Private animal health service provider (reference level)	PERG PERC PERP
Administration cost (UGX per animal) *	High (500) Medium (350) Low (200)	ADMCOST (Continuous)
Price of vaccine (UGX per animal) *	1,200 1,000 800	PRICE (Continuous)

^*^The currency conversion at the time of study was 1USD=UGX 3700, UGX = Uganda shillings.

#### 2.1.3 Generation of experimental design

This study followed the procedure laid down by ChoiceMetrics (27), in designing the choice experiment for this study. Following Masemola et al. (23) and Otieno et al. (28), a pilot study was conducted to generate prior coefficients and test the study instruments on 18 farmers. To determine the final design, the study followed ChoiceMetrics (2018), where the utility function for the likely model was stated. In this study, all the parameters were assumed to be generic and not alternative specific. Attributes that were to be estimated as dummy effects (discontinuous) were specified; these included time, quality of vaccinator proof of vaccination certificate and personnel vaccinating. The parameters from the pilot study were used and the multinomial logit model was stated as the econometric model that was likely to be estimated from data collected using the experimental design. For the price and administrative costs, the figures from these levels were used in the design matrix.

In line with Caussade et al. (29), a row-based algorithm using fractional factorial was used to determine the design with low efficiency errors as possible. The design selected had a *D*-error of 0.258 and *A*-error of 0.478. The 36 generated choice tasks were randomly divided into six blocks (ChoiceMetrics 2018). Each district represented a stratum and from a total sample of 148 respondents from each stratum, at least 24 were randomly allocated to each of the six blocks. To avoid bias and fatigue each farmer was asked to respond to only one block containing six choice sets. Each choice task had two unlabeled alternatives and an option “will *not buy*.”

#### 2.1.4 Choice set presentation

**Figure 1:**
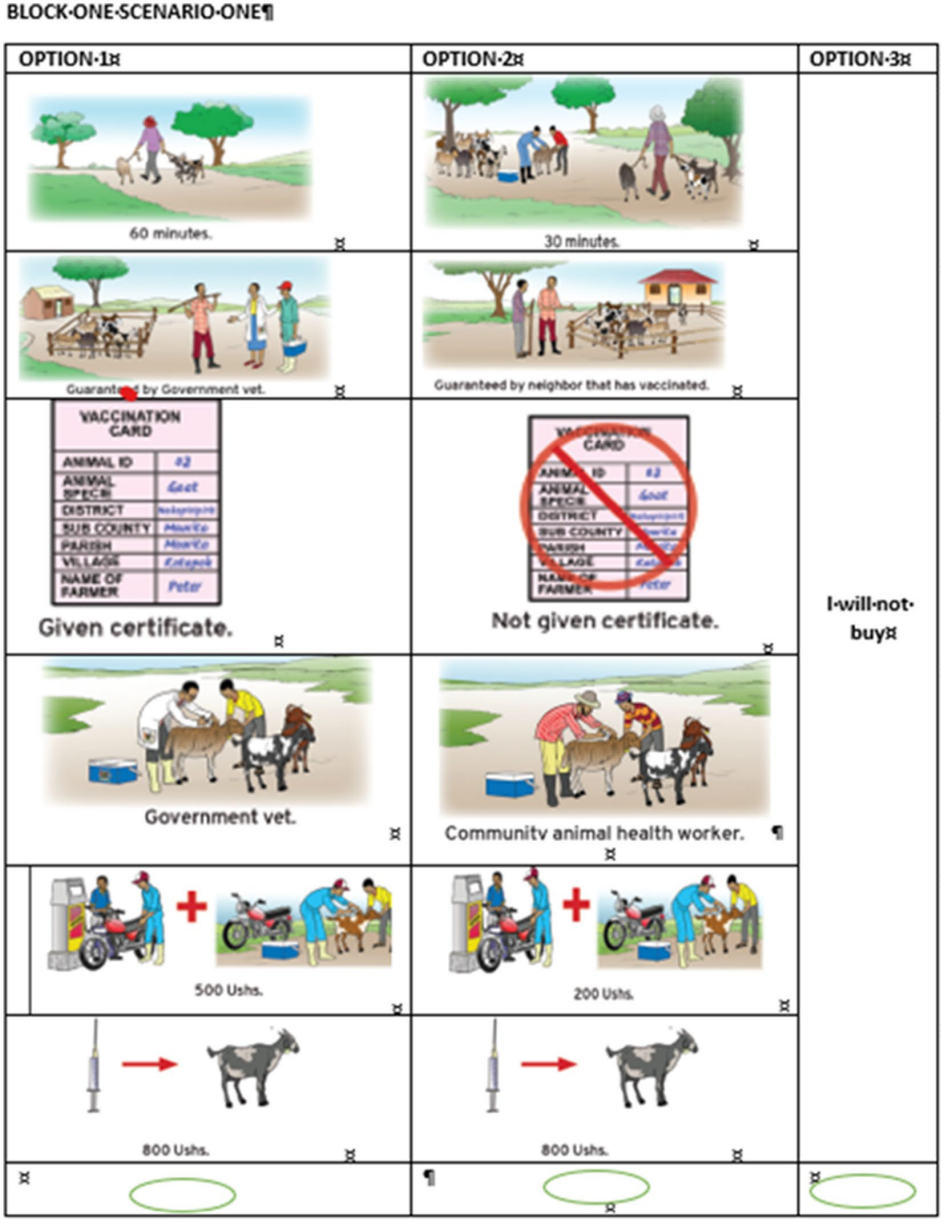
Pictorial form of the choice task.

Before the farmers were presented with the choice tasks, a cheap talk script adopted from Tonsor and Shupp (30), was used to limit hypothetical bias. The cheap talk script that was read out to each farmer was states as: *“The experience from previous similar surveys is that people often do not buy the goods when they are brought to the market. For instance, a recent study asked people whether they would purchase a new vaccine like the one you have been asked most indicated that they would buy but when the vaccine was stocked almost all of them did not buy. Accordingly, it is important that you make each of your upcoming selection of choices like you would if you were going to buy the vaccine in real life or taking your animals for vaccination, noting that vaccination of your animals will reduce on the money available for other purchases. Please look critically and make a choice.”* The tasks were presented to the farmers in a pictorial form (Figure 1). This allowed even farmers with low literacy levels to fully participate. In each choice situation, each farmer was asked to choose between the two vaccine alternatives or the option ‘I would not buy any of these vaccines’.

### 2.2 Study sites

**Figure 2:**
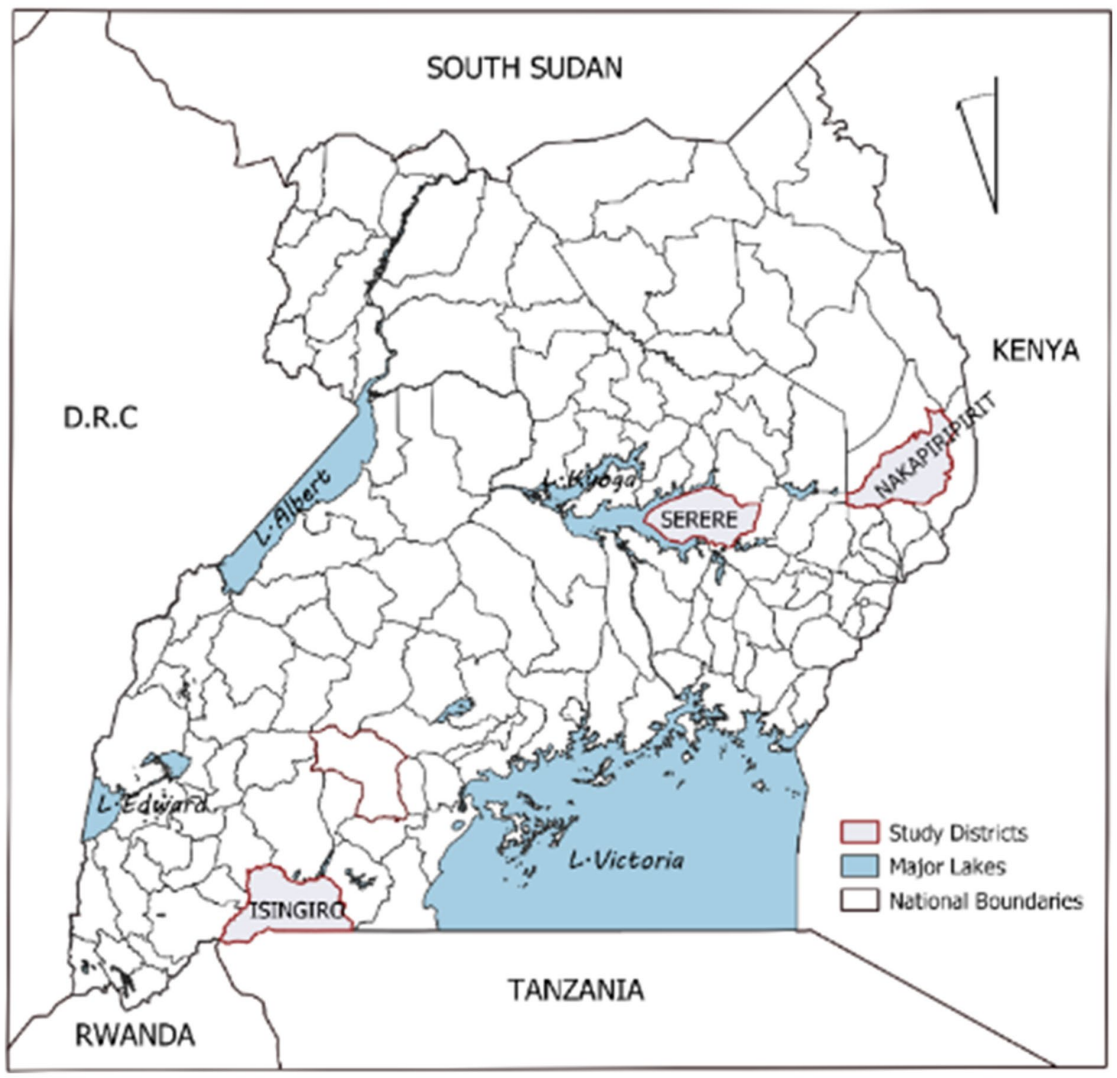
Study site generated using QGIS 3.22.

Study site selection was based on small ruminant populations, production systems and risk of PPR outbreaks. The characterization of the livestock production system (pastoral, agropastoral and mixed) was based on Cecchi et al. (3) that uses GIS (global information system) layers of human and livestock populations, length of growing period and land cover combined with the livelihood-based mapping enables regional comparison particularly within the East African region. In Uganda, within the cattle corridor, there are 21, 20 and 11 districts for agro-pastoral, mixed and pastoral livestock production systems, respectively, refer to Nkamwesiga et al. (12). All these districts have distinct characteristics that make them unique to fit the production system. From each production system one district was purposively selected for empirical inference. These districts included; Nakapiripirit, Serere and Isingiro (Figure 2). The risk of PPR outbreaks was based on the number of occurrences in previous year (31). Thus, districts were classified as high, medium and low risk. From the above criteria, Nakapiripirit was classified as a pastoral system with 1.2 million small ruminants and high risk to PPR outbreaks (2, 31). Isingiro was classified as an agro-pastoral livestock production system with150,000 small ruminants and low risk of PPR outbreaks. Lastly, Serere was classified as a mixed crop-livestock production system with 280,000 small ruminants and low risk to PPR outbreaks (2, 31). A multistage sampling method was employed. Three smaller administrative units (sub-counties) within each district were randomly selected. From these sub-counties, two lower administrative units (parishes) were also randomly selected. Using the Bartlett et al. (32), a sample size of 385 farmers was generated. To cater for dropouts, since the study was carried out in some pastoral communities, a rate of 15% was assumed giving rise to a sample size of 444. A list of all farmers within the parishes was generated from the district livestock production office. These were entered in excel and a random sample of 444 farmers was generated and interviewed.

### 2.3 Model specification

The choice made by the farmer was modeled as a function of attributes using random utility theory, which states that individuals will make choices based on the characteristics of a good (an objective component) along with some degree of randomness (a random component). This utility is represented in a discrete choice model by a general utility expression represented in Equation 1.


$$U_{\mathrm{ij}}=V_{\mathrm{ij}}+\varepsilon_{\mathrm{ij}}$$ (1)

Where $U_{\mathrm{ij}}$ is the unobserved utility that farmer *i* obtains from choosing j vaccine; $V_{\mathrm{ij}}$ is the systematic component that is a function of observable attributes and $\varepsilon_{\mathrm{ij}}$ is the random component.

To fit the utility function stated above within a discrete choice framework, the attributes $V_{\mathrm{ij}}$ must be mutually exclusive from the individual *i* perspective. Choosing one attribute necessarily implies not choosing any of the others. Second, the attributes (choice sets) must be exhaustive, in that all possible attributes are included and the individual chooses one of the attributes. Third, the number of attributes must be finite; the researcher can count the alternatives until they are exhausted (33).

In evaluating choice data, probit and logit models are often used. Probit and logit models do not represent different utility structures, but normally differ in the assumptions made for the error term, with one assuming a normal distribution, while the other assumes a multivariate generalized extreme value distribution of three types: Gumbel, Frechet and Weibull. In the probit, the variance of the unobserved effects is normalized to one such that the scalar value is equal to the square root of 1.5 while in the logit model the scalar value is normalized to one, implying that the coefficients of the logit model will be between 1.3–1.5 higher than those of the probit in a similar model. The reason for not choosing the probit is that it is much more cumbersome to compute, even with modern computer software. This study applied logit models. Conventionally, choice models have been applying the conditional logit model (CL), also commonly known as the multinomial logit (MNL). In this model, we make the assumption that the error terms are independently and identically distributed (Gumbel). Theoretically, the MNL is stated as Hensher et al. (34) and represented in Equation 2,


$$U_{\mathrm{ij}}=V(x_{\mathrm{ij}},s_{i})+\varepsilon_{\mathrm{ij}}$$ (2)

This can be further expanded to represent the different parameters to be estimated as presented in Equation 3


$$V(x_{\mathrm{ij}},s_{i})=\sum_{k=1}^{K}\beta_{\mathrm{ik}}x_{\mathrm{ijk}}\;$$ (3)

Where:

$x_{\mathrm{ijk}}$ is the level of attribute *k* for alternative *j*, $\beta_{\mathrm{ik}}$ the corresponding utility coefficient. Using the standard logit method, the probability that individual *i* will select an alternative *m* in the *T* sequence of choices is Equation 4:


$$Pr_{\mathrm{im}}=\Pi_{t=1}^{T}\frac{e^{\sum_{k=1}^{K}\beta_{\mathrm{ik}}x_{\mathrm{ijk}}}}{\sum_{j=1}^{J}e^{\sum_{j=1}^{J}\beta_{\mathrm{ik}}x_{\mathrm{ijk}}}}$$ (4)

This method has two limitations; it assumes that all respondents are the same across the sample, thus not accounting for preference heterogeneity. It also assumes that the difference in the scale of preference weights is constant across individuals, thus not considering scale heterogeneity (34). This has led to extensions of the model to address these limitations. Latent class (LC) and random parameter (RPL) models are alternatives to MNL that have been widely applied. The former assumes discrete distributions that define the latent structures of preferences, while the latter assumes a distribution of preference weights and models the parameters of that distribution for each attribute (35). For the derivations of the RPL and LC and their extension latent class random parameter (LCRP) the authors referred to Hensher et al. (34). To estimate the marginal rate of substitution commonly known as willingness to pay, the following Equation 5 was applied Hensher et al. (34).


$$\mathrm{WTP}=-\frac{\beta_{\mathrm{ik}}}{\beta_{i0}}$$ (5)

Where, $\beta_{\mathrm{ik}}$ is the estimated coefficient for an attribute level and $\beta_{i0}$ is coefficient of the price.

Lastly, a compensating surplus (CS) (the amount of money needed to return a small ruminant producer to their original level of utility after a vaccine price change) was calculated for the different cost scenarios (Equation 6):


$$\mathrm{CS}=\frac{-1}{\beta_{i0}}(V_{1}-V_{0})$$ (6)

Where $V_{1}$ is the value of the indirect utility associated with attributes in the policy scenario whereas $V_{0}$ is that of the baseline.

### 2.4 Data analysis

When coding the data, dummy coding (denotes the existence of a particular attribute with a one and its absence with a zero) was used rather than effects coding (the base level receives a value of minus one (−1) for each of the newly constructed effects coded variables). Although effects coding is preferred Hensher et al. (34), recent flaws that have been indicated in the interpretation of results (36). This misinterpretation normally leads to conflicting preference ordering, thus invalidating marginal willingness to pay estimates (36). This is especially true for those categorical attributes with more than two levels. Since this study had such attributes, dummy coding was applied.

The MNL was used as a starting model for estimating the RPL and LCRP. The RPL estimated in order to determine the sources of heterogeneity thus enabling to test the overall hypotheses. The RPL was chosen for analysis because it was expected that the likelihood of farmers choosing an alternative from the deterministic component could affect their preferences. Secondly since farmers were responding to six cards, RPL could accommodate the panel nature of the data. Thirdly, it relaxes the restrictive assumption of independence of irrelevant alternatives. Lastly, it accounted for heterogeneity in the data.

The RPL was estimated following the steps that are suggested by Hensher et al. (34). Exploratory analysis was done for both the distribution and heterogeneity. The first step was to run the model assuming that all the attributes were random with normal distribution. Then the next step was to re-specify the model with other distributions to find if the non-random parameters would be random. Lastly the final model was estimated by specifying the insignificant attributes as non-random with the distribution that had the best model fit (triangular distribution). For the willingness to pay (WTP), confidence intervals were calculated from the standard deviations using the delta method for the attributes that had heterogeneity in the means while for the others, the point estimation was used (34). Only the significant attributes were used in calculating the WTP (37). The overall WTP or the compensating surplus (CS) welfare measure was obtained for different scenarios associated with changes in attribute levels. This was to measure the response of farmers to different attributes.

For comparison, the study went further to run the LCRP to intuitively assess whether there was discrete distribution of the data by classes and to further assess if all the heterogeneity was explained by the classification. The LCRP was estimated in two steps; the first step was to run the LC to identify the classes, and then later its extension LCRP was run to check whether the classes accounted for all the heterogeneity among the attributes. Analysis was done using NLOGIT 6 software.

## 3 Results

### 3.1 Farmer characteristics

The prior classification of the study areas into pastoral (Nakapiripirit), agro-pastoral (Isingiro) and mixed crop-livestock systems (Serere) was supplemented by additional variables that were collected to check if the categorization suited the data. These variables included the feeding system and the share of small ruminant income in overall income (measured as the ratio of the income from small ruminant products to the total household income in the last 12 months) (38, 39). From the results below (Table 2), it was observed that in Nakapiripirit the predominant feeding system was open grazing while that of Serere was tethering. Furthermore, Isingiro had almost equal proportions of farmers that tethered and open grazed their small ruminants. This justified categorizing the areas by production system. Nakapiripirit had significantly higher TLUs than the other two districts. The Isingiro households had a significantly higher average annual income. The results on TLUs and income reaffirm the national statistics that were reported within these areas. Nakapiripirit had significantly lower levels of household heads who had education above upper primary. Serere had the lowest number of farmers who were aware of PPR disease (farmers were asked if they were aware of the name of the disease in their local language). Although many farmers were affected by livestock diseases, few had performed vaccinations. This indicates that vaccination is not widely used as a disease prevention mechanism. It could also be indicative of limited knowledge of vaccines or specific vaccines for specific diseases. There was even low membership in the groups that carried out livestock production in the three districts. When the variance analysis was performed across these production systems, it was found that some variables had significant differences between means (Table 2). A Bonferroni test was performed to determine which production systems were different.

**Table 2:** Characteristics of the sampled farmers.

Characteristic	Nakapiripirit	Isingiro	Serere	Pooled
	*n =* 148	*n =* 148	*n =* 148	*n =* 444
Average years in SR production	11.9^a^	17.8^a^	22.8^a^	17.5
Average household size	5.5	5.5	5.1	5.4
Average land size in acres	5.8	3.9	4.1	4.6
Average TLUs	12.6^ab^	4.3^a^	6.1^b^	7.7
Average age	40.9^ab^	49.5^a^	51.9^b^	47.4
Average total annual income (UGX)*	898,203.5^a^	1,421,568.0^ab^	692,101.4^b^	989,205.2
Average total annual income (USD)*	256.2	384.2	187.1	392.0
Education in years	1.8 ^ab^	5.5 ^a^	6.3 ^b^	4.6
Ratio of small ruminant income to total income	0.13^a^	0.26^ab^	0.17^b^	0.19
Education above upper primary (%)	14.86	49.32	67.57	43.92
Livestock activity group membership (%)	17.57	16.89	9.46	14.64
PPR awareness (%)	66.89	46.62	10.14	41.22
Vaccinated livestock in the last 12 months (%)	25.0	29.73	33.11	29.28
Affected by livestock diseases in last 12 months (%)	83.11	76.35	71.62	77.03
Male respondents (%)	62.84	56.76	66.89	62.16
Feeding type (%)				
Open grazing	0.00	49.32	90.54	46.62
Tethering	100.00	50.68	9.46	53.38

^a,b,c^Represents statistical differences with Nakapiripirit, Isingiro, and Serere, similar letters indicate differences between groups within a row. * 1USD=UGX 3700.

### 3.2 Empirical analysis

#### 3.2.1 Random parameter logit results

From the pooled sample (Table 3), all attributes were significant compared to their reference levels, except the quality attribute level that public animal health workers assure. Price and administrative costs were both significant and had negative signs, in accordance with economic theory, although their coefficients were too low compared to other attributes. Additionally, the time was negative and significant. This indicated that lower prices, administration costs, and less time to vaccination centers were preferred. Farmers preferred the assurance of their local leaders of quality vaccines more than the assurances of their neighbors. When it came to vaccination, farmers preferred community animal health and government vaccinators compared to private animal health vaccinators. Farmers preferred to get a certificate after vaccination to allow them to move their animals. This attribute was highly significant and had a remarkably large coefficient compared to other attributes.

**Table 3:** Random-parameter logit results for vaccine delivery attributes.

Attributes	Coeff	(SE)
Random parameters in utility functions		
Given certificate	1.059***	0.127
Price	−0.001***	0.000
Nonrandom parameters in utility functions		
Time to vaccination center	−0.007***	0.002
Local leader assures quality	0.134*	0.076
Public animal health worker assures quality	0.048	0.082
Government animal health vaccinator	0.479***	0.085
Community animal health vaccinator	0.288***	0.088
Administration costs	−0.007***	0.000
Diagonal values in Cholesky Matrix (triangular)		
Given certificate	1.809***	0.241
Price	0.002***	0.000
Below diagonal values in L matrix		
Price: Given certificate	0.001**	0.001
Standard deviations of random parameters		
Given certificate	1.673***	0.249
Price	0.003***	0.000
Covariances of random parameters		
Price: Given certificate	0.002***	0.000
Log-likelihood	−2125.582	
Adjusted pseudo-*R*^2^	0.274	
Halton draws	100	
N (respondents)	444	
N (observations)	2,664	

Mean shifters	Coeff	(SE)
Given certificate		
Open graze	−0.237	0.151
PPR awareness	0.141	0.147
Flock size		
Vaccinated	−0.046	0.145
Advisory services	−0.039	0.159
Income ratio	0.392	0.239
Farmer group membership	0.070	0.200
Price		
Open graze	0.001***	0.000
PPR aware	−0.000	0.000
Flock size	0.000	0.000
Vaccinated	−0.000	0.000
Advisory services	0.000	0.000
Income ratio	0.000	0.000
Farmer group membership	−0.001***	0.000

***, **, * Significance at the 1, 5, 10% level.

Heterogeneity in the means was exhibited in certification and price indicated by the statistically significant standard deviations. This shows that farmers had very diverse perspectives or opinions on these two attributes. But for all other attributes, the mean coefficients were enough to explain the sample farmers. To test the hypothesis of significant differences within production systems, the interaction effects of key variables were included in the RPL. These included variables of the feeding system (tethering and open grazing) and the small ruminant income ratio. The tethering was used as the reference variable for the feeding system in the model. From the results, it could be observed that the heterogeneity of the price attribute could be explained by the feeding system. Farmers using the open grazing system were observed to be less sensitive to price changes than those who practiced tethering as a feeding system.

Further analysis was carried out for districts, as they represented the different production systems (Table 4). From the results in Table 4, there was a variation in significance between attributes in the different districts. Only preference for certification and administration costs was significant in all three districts. It was only in Nakapiripirit (a largely pastoral system) that the time to vaccination centers was significant. Furthermore, in Nakapiripirit, the price was insignificant, but administrative costs were significant. Where quality assurance was important (Nakapiripirit and Serere), the significance varied, with mixed production systems preferring local leaders, while the pastoral preferred government officials compared to quality being assured by the neighbor.

**Table 4:** Random parameter logit results for vaccine delivery attributes in different districts.

**Attribute**	**Nakapiripirit**		**Isingiro**		**Serere**	
	**Coeff**	**(SE)**	**Coeff**	**(SE)**	**Coeff**	**(SE)**
Time to vaccination center	0.009**	0.004	−0.005	0.004	−0.005	0.233
Local leader assures quality	−0.047	0.120	−0.087	0.131	0.477***	0.129
Public animal health worker assures quality	0.313**	0.132	0.009	0.144	−0.204	0.181
Given certificate	0.554***	0.097	1.400***	0.110	0.902***	0.104
Government animal health vaccinator	0.354***	0.132	0.194	0.145	0.775***	0.141
Community animal health vaccinator	0.184	0.141	−0.181	0.155	0.711***	0.150
Administration costs	−0.001**	0.000	−0.001*	0.001	−0.001*	0.000
Price	−0.000	0.000	−0.002***	0.000	−0.001***	0.000
Random parameter standard deviations						
Time	0.022*	0.132	0.002	0.023	0.000	0.013
Given certificate						
Price	0.003***	0.001				
Log-likelihood	−704.210		−672.367		−701.342	
Adjusted pseudo-*R*^2^	0.278		0.311		0.281	
Halton draws	100		100		100	
N (respondents)	148		148		148	
N (observations)	888		888		888	

***, **, * Significance at the 1, 5, 10% level.

The price attribute was used to determine the WTP values for farmers in the pooled sample, district of Serere and Isingiro (Table 5). For Nakapiripirit, the WTP values were not calculated because the price attribute was insignificant (see Table 4). Statistical differences between districts could not be identified because different attributes were significant in different districts. From the pooled sample (Table 5), farmers were willing to pay the highest for certification.

**Table 5:** Marginal WTP for vaccine attributes (UGX) (1 USD = 3,700 UGX).

Attribute	Marginal WTP (95% confidence interval)		
	Pooled	Isingiro	Serere
Time	−4 (−421 to 412)		
Local leader assures quality	88 (−8,147 to 8,323)		441
Given certificate	937 (−45,360 to 47,234)	902 (902 to 902)	835 (−70 to 1,941)
Government animal health vaccinator	315 (−29,181 to 29,812)		717 (−664 to 3,058)
Community animal health vaccinator	189 (−17,7,549 to 17,928)		658 (−856 to 2,585)

Compensating surplus (CS) estimates were derived for two possible scenarios and only for the pooled sample (Table 6). The first scenario included the government subsidizing the cost of vaccine administration. This was considered a feasible scenario, since most vaccinations are done by government vaccinators. The second scenario was one in which the government could provide the vaccine at a reduced cost. Positive CS for both scenarios indicated that farmers preferred improved attribute levels. It was observed that the scenario with no administration costs had a higher CS (Uganda shillings (Ugx) 387) compared to reduction in price Ugx 251.

**Table 6:** Compensation surplus estimates for vaccine delivery policy scenarios (UGX*).

Attribute	Scenario	
	No administration cost	Price reduction
Local leader	X	X
Public animal health worker	X	X
Given certificate	X	X
Public animal health service provider	X	X
Community animal health worker	X	X
Administration costs	0	100%
Price	100%	50%
Compensating surplus		
Mean	387	251
Std. dev.	434	434

*UGX, Uganda shillings.

#### 3.2.2 Comparison between random parameter and latent class random parameter estimates

The LC with three classes better fitted the data; the results are presented in Table 7. Of the three classes, farmers were more likely to be in class one than in the other two classes, as indicated by the higher probability. For the LCRP, all the heterogeneity in the means was taken into account by the classes. This could be observed with the statistical insignificance of all the standard deviations in Table 7. Considering class heterogeneity determinants, significant coefficient of a variable in the class was interpreted as an increased probability that a farmer with this characteristic would be in that class rather than in class three (which was omitted). For example, open-graze farmers were more likely to be in class one than in class three. Similarly, farmers with a high-income ratio and aware of PPR but with fewer flocks were more likely to be in class two than in class three. From Table 7, price was significant in classes two and three, but not in class one (though with the expected sign). Administrative costs were significant in classes one and three.

**Table 7:** Results of the latent class random parameter model on the attributes.

Attributes	Class one		Class two		Class three	
	Coeff	(SE)	Coeff	(SE)	Coeff	(SE)
Time to vaccination center	−0.009***	0.002	0.006	0.008	0.0231*	0.013
Local leader assures quality	0.137	0.096	−0.858*	0.466	2.473***	0.572
Public animal health worker assures quality	0.335***	0.114	−1.013	0.623	−2.886***	0.637
Given certificate	0.49369***	0.091	4.146***	0.740	3.106***	0.679
Government animal health vaccinator	0.526***	0.110	−1.101*	0.602	2.028***	0.581
Community animal health vaccinator	0.270**	0.110	−1.962**	0.769	2.241***	0.656
Administration costs	−0.001***	0.000	−0.001	0.002	0.003*	0.001
Price	−0.000	0.000	−0.006***	0.001	−0.005***	0.002
Standard deviations of random parameters						
Time to vaccination center	0.000	0.003	0.000	0.009	0.000	0.010
Local leader assures quality	0.001	0.153	0.000	0.448	0.001	0.459
Public animal health worker assures quality	0.001	0.151	0.003	0.477	0.001	0.524
Given certificate	0.001	0.137	0.001	0.446	0.004	0.464
Government animal health vaccinator	0.000	0.149	0.005	0.474	0.001	0.473
Community animal health vaccinator	0.002	0.146	0.007	0.395	0.001	0.497
Administration costs	0.000	0.000	0.000	0.001	0.000	0.001
Price	0.000	0.000	0.000	0.000	0.000	0.001
Mean shifters in classes						
Open graze	2.362***	0.802	1.892**	0.840		
PPR aware	0.887	0.640	1.381**	0.667		
Flock size	−0.017	0.014	−0.048*	0.026		
Vaccinated	−0.279	0.463	−0.079	0.524		
Advisory services	−0.039	0.439	−0.600	0.568		
Income ratio	0.508	0.817	1.911**	0.842		
Farmer group membership	0.463	0.678	0.679	0.773		
Class probability	0.647		0.209		0.145	
Number of respondents (N)	285		92		67	
Nakapiripirit (N)	129		16		3	
Isingiro (N)	87		57		4	
Serere (N)	69		19		60	
Log-likelihood	−2074.040					
Adjusted pseudo-*R*^2^	0.291					

***, **, * Significance at the 1, 5, 10% level.

Like in the RPL, all classes had a significant preference for obtaining certificates. An important result to note are the differences between the results in Tables 4, 7. It could be observed that with the hypothetical split of farmers by district that lumped all farmers in one cluster based on assumed production systems (RPL), the LCRP reconstituted these farmers and distributed them in different classes (Table 8). For example, of the 77% of the farmers from the pastoral livestock production system (Nakapiripirit) were in class one Furthermore, 52% of the farmers from the agro-pastoral system (Isingiro) fit in class two while 84% of the farmers in the mixed production system were in class two and three. The significant variables that accounted for the heterogeneity varied between the two models. Differences in preference weights and their comparison in the RPL and RPLC is an empirical question (Boeri et al., 2020). From the results, it is concluded that the LCRP fits the data slightly better than the RPL (see Table 3 for comparison). The LCRP had a slightly lower logarithmic likelihood and a higher *pseudo-R^2^*.

**Table 8:** Percentage of respondents in different classes and livestock production systems.

Latent classes	Pastoral	Agro-pastoral	Mixed
Class one	77.7	30.4	15.5
Class two	17.6	52.7	41.2
Class three	15.5	16.9	43.2

## 4 Discussion

The results of this study demonstrated the existence of heterogeneity in certification and price. The other attributes were homogenous across all the production systems. The study expected time to vaccination center to vary given the diverse livestock production system but this did not vary. This indicates designated places for vaccinations are not favorable across all production systems. Certification was an important issue in all production systems, probably because it was tied to the ease of movement of animals during quarantine for goat plague. This is in line with the study by Ilboudo et al. (40), who noted that certification and vaccinating personnel were the greatest influencers on whether farmers would vaccinate their animals. Furthermore, Gethmann et al. (41), indicated that the main driver of farmers participating in vaccination was the ability of them to sell their animals. The low-price coefficients compared to other attributes could be explained by the minimal amount of the vaccine price compared to the total value of sheep and goats. For example, a mature goat can have a market price of approximately Ugx 200,000 (USD 54) compared to the vaccine price of approximately Ugx 1,000 (USD 0.27). This made other attributes, such as vaccination certificates and the movement of animals to vaccination centers more important attributes. Several authors for instance, Iles et al. (42) and Masemola et al. (23) have reported similar trends in the price attribute on vaccine preferences.

In this study it was reported that farmers preferred vaccination closer to home especially in the pastoral areas (Nakapiripirit) could be due to the difficulty of controlling small ruminants for long distances if they were to trek to the vaccination centers. Given the expanse of the pastoral areas, another reason could be that the vaccination centers are located quite far for most farmers compared to the other production systems. This compares with Ilboudo et al. (40), who indicated farmers’ preference to vaccinate at home to minimize costs associated with movement of their animals. Furthermore, since women are known to be responsible for small ruminants and sick animals in pastoral areas De Jode and Flintan (43), the inconvenience of trekking the animals could have had a major effect. For Nakapiripirit (pastoral), the price was insignificant, but administrative costs were significant. This reaffirms Acosta et al. (44) findings that in pastoral communities of Uganda, vaccines provided are always subsidized by the government and non-government organizations but farmers must cater for the administration costs. In areas that lack this subsidy like Serere (mixed production system) price was significant.

From the above results, it could be deduced that policy makers from both local and central governments could apply different strategies for different production systems. For example, in pastoral areas where time to vaccination centres was important, vaccinations could be carried out within the villages of the farmers. This would increase the turn-up rate and thus improve the coverage. There is also a need to peg vaccination with certification, as this could improve the success of vaccination campaigns. The authors appreciate the challenge of implementing this given the lack of proper animal identification system in the country, nevertheless the results indicated a justifiable need. There is a need for awareness campaigns on the quality and safety of vaccines and in pastoral areas, policy makers could use government officials, since they are trusted, while in mixed crop-livestock production systems local leaders could be used in quality campaigns. Across all production systems, farmers trusted their local and government animal health vaccinators. This poses a challenge if the vaccine were to be availed as a private good and yet private veterinary practitioners are more readily available than the government. The best solution would be to make farmers aware that all the private animal health service providers are supervised by the district veterinary officer. This would give confidence to the farmers.

In regards to WTP for the vaccine, it could be concluded that in both Serere and Isingiro districts, farmers were willing to pay for the vaccine, unlike Nakapiripirit. This would mean that the promotion of vaccine as a private good could be concentrated in these two districts, while subsidies could be continued in Nakapiripirit. An administration cost subsidy could be implemented across all districts, while a price subsidy could be applied to Nakapiripirit. If the government wishes to implement subsidies, an administration cost subsidy will be much more effective given its high compensating surplus. This can be implemented by government subsidizing the cost of the vaccinator, while farmers can offer to restrain their animals. As indicated earlier subsidizing of the vaccine has been taking place in the pastoral communities.

When comparing the RP and LC model it can be seen that the LCRP that both models gave insights to the heterogeneity of preferences. On the high level of the whole sample, the RP was able to indicate the heterogeneous attributes while the LCRP was able to subgroup the sample into clusters. While the RP was used to further look for the sources of heterogeneity at the predefined districts that were representing the production systems, the LCRP was able to statistically distinguish the sample into groups that were slightly different. This indicates that although farmers can be clustered in one stratum like a district and generally assumed to be homogeneous, there are some farmers that are likely not fitting in the cluster. When comparing the attributes that accounted for heterogeneity among the two models, it can be seen that while in the RPL only one variable (feed system) was significant, in the RPLC several variables accounted for heterogeneity in the classes.

## 5 Conclusions and policy recommendations

This study analyzed preferences for the delivery of PPR vaccines in Uganda in different production systems based on district categorization. Before this study, limited evidence had been presented on the preferences of vaccine delivery systems for PPR and the farmers’ WTP for the PPR vaccine in Uganda. The study established that there was variations among small ruminant farmers in regards to vaccine price and certification. The study demonstrated that different strategies could be taken when carrying out vaccination in line with Uganda’s PPR eradication strategy. First strategy was availing the vaccine to the production systems that were willing to pay (agro-pastoral and mixed production systems) and providing cost subsidization for the pastoral communities with the government providing the vaccine while the farmers catered for some of the vaccine administration costs like restraining of the animals. The second strategy would be to subsidize administration costs across all production systems (since this was significant across all districts). This could be implemented by government provision of the vaccinator through their extension system. This would also be beneficial since farmers indicated that these vaccinators were the most trusted. The third strategy could be to pair vaccination with certification. The authors appreciate the challenge of implementing this given the lack of proper animal identification system in the country, nevertheless the results indicated a justifiable need. It is also highlighted by the Uganda national strategy for PPR eradication for identifying a system that can differentiate the vaccinated from the non-vaccinated animals. The other option would be to ear notch all the vaccinated animals in a way identifiable by the government thus eliminating the farmers need for carrying certificates. The study recommends that vaccination should be performed in farm communities, especially in pastoral communities. This would increase the turn-up rate and thus improve the coverage. The study recommends building the capacity of community animal health workers to carry out vaccinations. This, in turn, would bring the vaccination service closer to communities. The study also recommends the use of local leaders and government agencies in mixed and pastoral production systems, respectively, to create awareness of vaccination campaigns, as they are the most trusted.

### 5.1 Limitations of the study and suggestions for further research

Though the study sampled in all the production systems, there is need to increase the number of districts sampled in each production system in order to improve the generalizability of the findings. Secondly, the study used a hypothetical design (participants talked about hypothetical money in their pockets) compared to real-life scenarios in which farmers would receive money and actually buy the desired vaccine option. Where resources permit, future studies could offer more practical insights by providing money to enable respondents mimic real life market situations. Thirdly, the chosen parameter structure may not fully capture heterogeneity across all relevant attributes, which in turn affects the interpretation and generalizability of the findings. Lastly, some of the recommendations would require government implementing a national animal identification system that would require considerable resources. Therefore, there is need for further empirical studies on the priorities and acceptance of other stakeholders on the attributes analyzed in this study. Moreover, the economic feasibility of various policy scenarios and resource mobilization by different actors require further consideration in research.

## Data Availability

The raw data supporting the conclusions of this article will be made available by the authors, without undue reservation.
